# Sex, Atrial Fibrillation, and Long-Term Mortality After Cardiac Surgery

**DOI:** 10.1001/jamanetworkopen.2024.26865

**Published:** 2024-08-21

**Authors:** Sergey Karamnov, Natalia Sarkisian, Jakob Wollborn, Samuel Justice, Kara Fields, Vesela P. Kovacheva, Asishana A. Osho, Ashraf Sabe, Simon C. Body, Jochen D. Muehlschlegel

**Affiliations:** 1 Department of Anesthesiology, Perioperative and Pain Medicine, Brigham and Women’s Hospital, Harvard Medical School, Boston, Massachusetts; 2 Department of Sociology, Boston College, Chestnut Hill, Massachusetts; 3 Division of Cardiac Surgery, Brigham and Women’s Hospital, Harvard Medical School, Boston, Massachusetts; 4 Division of Cardiac Surgery, Massachusetts General Hospital, Harvard Medical School, Boston; 5 Department of Anesthesiology, Boston Medical Center, Boston University School of Medicine, Boston, Massachusetts; 6 Department of Anesthesiology and Critical Care Medicine, Johns Hopkins University School of Medicine, Baltimore, Maryland

## Abstract

**Importance:**

There are limited data on the association of sex with the incidence of postoperative atrial fibrillation (poAF) and subsequent long-term mortality after cardiac surgery.

**Objective:**

To evaluate whether the incidence of poAF and associated long-term mortality after cardiac surgery differ by sex.

**Design, Setting, and Participants:**

This retrospective cohort study was conducted at 2 tertiary care centers in Massachusetts from January 1, 2002, until October 1, 2016, with follow-up until December 1, 2022. Adult (aged >20 years) women and men undergoing coronary artery bypass graft surgery, aortic valve surgery, mitral valve surgery, and combined procedures with cardiopulmonary bypass were examined using medical records. Patients who had data on poAF were included in data analyses.

**Exposures:**

Sex and poAF.

**Main Outcomes and Measures:**

Primary outcomes were the incidence of poAF and all-cause mortality. poAF was defined as any atrial fibrillation detected on electrocardiogram (EKG) during the index hospitalization in patients presenting for surgery in normal sinus rhythm. Data on poAF were obtained from EKG reports and supplemented by information from the Society of Thoracic Surgeons Adult Cardiac Surgery Database. All-cause mortality was assessed via hospital records. The hypotheses were formulated prior to data analysis.

**Results:**

Among 21 568 patients with poAF data (mean [SD] age, 66.5 [12.4] years), 2694 of 6601 women (40.8%) and 5805 of 14 967 men (38.8%) developed poAF. In a multivariable logistic regression model, women had lower risk of poAF (odds ratio [OR], 0.85; 95% CI, 0.79-0.91; *P* < .001). During the follow-up study period, 1294 women (50.4%) and 2376 men (48.9%) in the poAF group as well as 1273 women (49.6%) and 2484 men (51.1%) in the non-poAF group died. Cox proportional hazards analysis found that the association between poAF and mortality was significantly moderated (ie, effect modified) by sex. Compared with same-sex individuals without poAF, men with poAF had a 17% higher mortality hazard (hazard ratio [HR], 1.17; 95% CI, 1.11-1.25; *P* < .001), and women with poAF had a 31% higher mortality hazard (HR, 1.31; 95% CI, 1.21-1.42; *P* < .001).

**Conclusions and Relevance:**

In this retrospective cohort study of 21 568 patients who underwent cardiac surgery, women were less likely to develop poAF than men when controlling for other relevant characteristics; however, women who did develop poAF had a higher risk of long-term mortality than men who developed poAF. This observed elevated risk calls for a tailored approach to perioperative care in women undergoing cardiac surgery.

## Key Points

**Question:**

Do the incidence of postoperative atrial fibrillation (poAF) and associated long-term mortality after cardiac surgery differ by sex?

**Findings:**

In a cohort study of 21 568 patients who underwent open heart surgery, controlling for patient and surgery-related risk factors, women had a significantly lower incidence of poAF than men, but poAF was associated with significantly higher adjusted mortality hazard in women compared with men.

**Meaning:**

These findings suggest that more vigilant monitoring and long-term follow-up care for women who develop poAF after cardiac surgery are warranted.

## Teaser

This cohort study evaluates whether the rates and outcomes of postoperative atrial fibrillation among patients undergoing open heart surgery differ by sex.

## Introduction

Women are at higher risk for postoperative morbidity and increased mortality after open heart surgery. In multiple studies, women had increased risk of postoperative congestive heart failure,^1^ sternal wound infection,^2,3^ kidney dysfunction,^4,5^ stroke,^1,6^ need for hospital readmission,^1^ and mortality.^7,8,9,10^

However, the association of sex with postoperative atrial fibrillation (poAF), the most common complication after cardiac surgery^11,12,13^—which is itself associated with stroke,^14,15^ long-term AF,^15^ and mortality^14,16,17^—remains unclear. Several studies have reported that men have greater risk of developing poAF,^18,19,20,21,30,31^ while others showed no difference in the incidence of poAF between men and women.^22,23,24,25,26,27^ While an insufficient overall sample size might explain this lack of association, other potential reasons could be the underrepresentation of women in study samples or focus on patients undergoing coronary artery bypass graft (CABG) only.^22,23,25,26,27^

Furthermore, it is unclear whether the link between poAF and long-term mortality differs by sex. That is, while the incidence of poAF may be higher in men (as some prior studies suggest), the strength of its association with long-term mortality may also differ by sex, possibly contributing to higher rates of overall long-term mortality among women despite their lower rates of poAF. Conflicting evidence has failed to provide clarity.^28,29^

To address this gap in understanding the role of sex in poAF and associated long-term mortality after cardiac surgery, we designed this multi-institutional retrospective cohort study. We hypothesized that (1) men are more likely to develop poAF than women and (2) the association of poAF and long-term mortality differs between men and women.

## Methods

### Ethics

The Brigham and Women’s Hospital institutional review board approval was obtained with a waiver of consent. This study follows the Strengthening the Reporting of Observational Studies in Epidemiology (STROBE) reporting guideline.

### Study Design and Population

**Figure 1.**
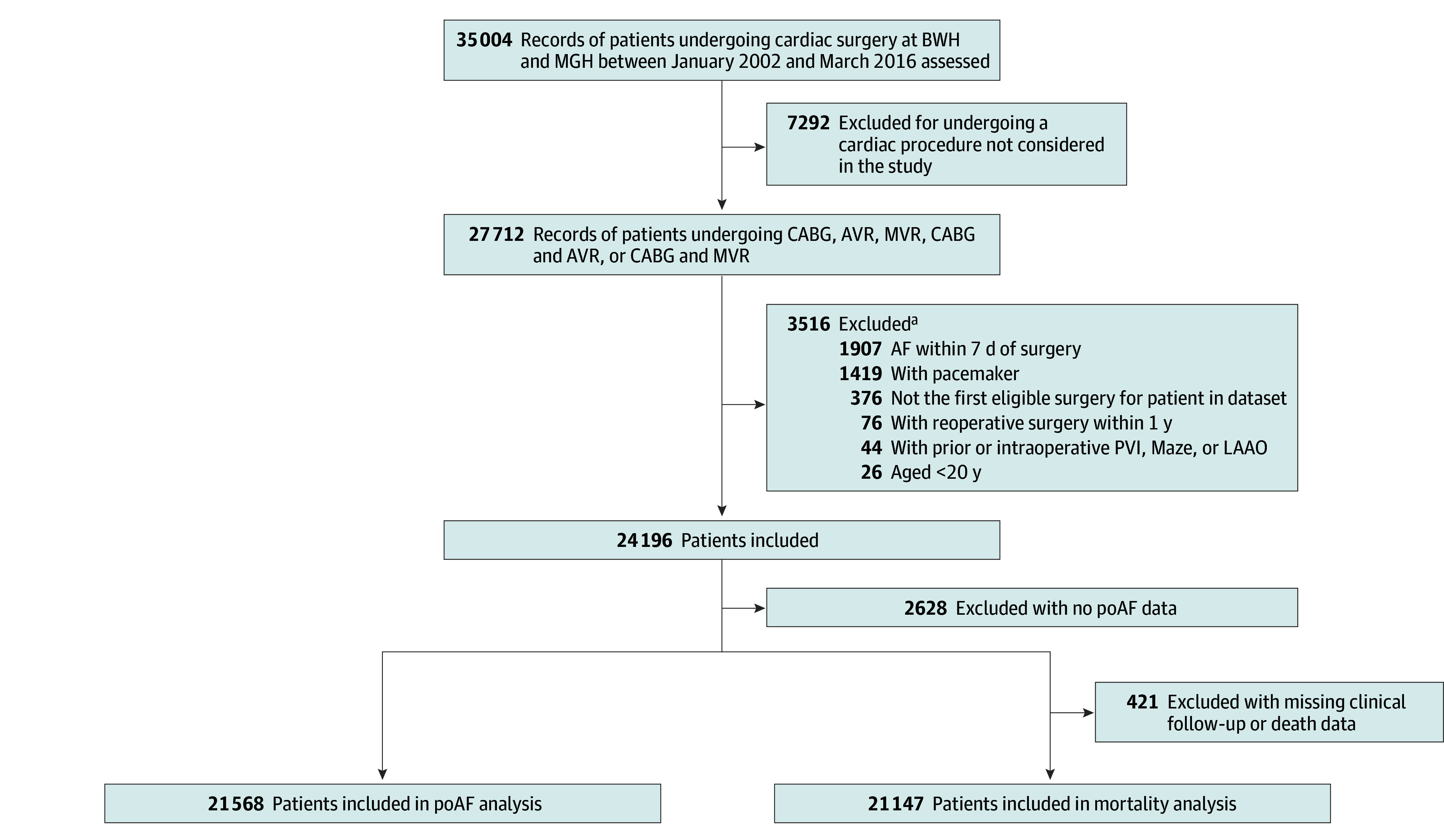
Flow Diagram of the Study With Inclusion and Exclusion Criteria AF indicates atrial fibrillation; AVR, aortic valve replacement; BWH, Brigham and Women’s Hospital; CABG, coronary artery bypass surgery; LAAO, left atrial appendage occlusion; MGH, Massachusetts General Hospital; MVR, mitral valve replacement or repair; and PVI, pulmonary vein isolation. ^a^Some records had multiple, overlapping exclusions.

This is a retrospective observational cohort study of adult (>20 years) patients who underwent CABG, open aortic valve replacement or repair (AVR), open mitral valve replacement or repair (MVR), or combined procedures (CABG and AVR or CABG and MVR) with the use of cardiopulmonary bypass (CPB) at 2 tertiary care centers. We obtained data from 2 hospital cohorts, Brigham and Women’s Hospital and Massachusetts General Hospital, using identical methods. Data for adult cardiac surgery patients who underwent primary open heart surgery with CPB between January 1, 2002, and October 1, 2016, with mortality follow-up until December 1, 2022, were obtained from the institutional Research Patient Data Registry,^30^ a comprehensive centralized clinical data registry that gathers and stores data from hospital systems while ensuring the security of patient information. We only included the first eligible surgery for a given patient in our analyses; subsequent procedures for the same patient were excluded. We included patients with a history of paroxysmal AF but excluded those with persistent or longstanding AF or those who were not in normal sinus rhythm within 7 days prior to surgery; we also excluded patients with pacemakers, those with prior or intraoperative pulmonary vein isolation, Maze, or left atrial appendage occlusion and those who underwent reoperations within 1 year. This set of exclusions (Figure 1) resulted in a dataset with 12 917 patients from hospital 1 and 11 279 patients from hospital 2.

### Measures of Outcomes

Primary outcomes were the incidence of poAF and all-cause mortality. We defined poAF as any AF that developed on electrocardiogram (EKG) during the primary hospitalization in patients who presented for surgery in normal sinus rhythm. This approach is consistent with the previously published literature.^12,31^ Additional data were extracted from Society of Thoracic Surgeons (STS) Adult Cardiac Surgery Database.^32^ This comprehensive database contains a longitudinal assessment of institutional outcomes after cardiac surgery, such as poAF.^12^ STS defines postoperative AF as AF that “lasts longer than one hour” or “lasts less than one hour but requires medical or procedural intervention.”^32^ We defined poAF was defined as the AF identified on the EKG or in the STS database.

Dates of birth, surgery, and death were obtained from hospital records that encompassed externally obtained mortality records. Censoring dates were defined as date of death or date of last medical record indicating patient being alive. Patients with no additional hospital encounters at the studied institutions beyond the index surgery and lacking institutional records of death were excluded from mortality analysis (1.9% of patients).

### Covariates

Covariates included patient demographics (age and race and ethnicity, which was self-reported and entered in the electronic health records as a single 5-category variable including Asian, Black, Hispanic, White and other [American Indian, Alaska Native, Native Hawaiian, or Other Pacific Islander; those who explicitly identified as other; and those who identified as 2 or more races]), patient-related risk factors (history of AF, hypertension, hyperlipidemia, history of myocardial infarction, congestive heart failure, peripheral vascular disease, diabetes, history of severe chronic obstructive pulmonary disease, body mass index [calculated as weight in kilograms divided by height in meters squared], and smoking status), surgery-related risk factors (procedure type, whether the surgery was emergent, and hospital), laboratory values (creatinine level, log-transformed to improve distribution), and medications (angiotensin-converting enzyme inhibitors or angiotensin II receptor blockers, β-blockers) (Table 1). To ensure linearity, we included square terms for age, body mass index, and the log of creatinine level (age and body mass index were divided by 10 prior to squaring to simplify presentation).

**Table 1.** Descriptive Characteristics of 21 568 Patients Older Than 20 Years Undergoing Open Heart Surgery With Cardiopulmonary Bypass From January 1, 2002, Through October 1, 2016

Characteristic	Participants by poAF status, No. (%)		*P* value for poAF^a^	Participants by sex, No. (%)		*P* value for sex^a^
	No poAF (n = 13 069)	poAF (n = 8499)		Women (n = 6601)	Men (n = 14 967)	
Sex						
Female	3907 (29.9)	2694 (31.7)	.005	NA	NA	NA
Male	9162 (70.1)	5805 (68.3)		NA	NA	
poAF	NA	NA	NA	2694 (40.8)	5805 (38.8)	.005
Age, mean (SD), y	63.8 (12.7)	70.6 (10.8)	<.001	69.0 (12.7)	65.4 (12.2)	<.001
BMI, mean (SD)	28.6 (5.7)	28.5 (5.8)	.24	28.6 (6.8)	28.5 (5.2)	.70
Race						
Asian	229 (1.8)	94 (1.1)	<.001	83 (1.3)	240 (1.6)	<.001
Black	307 (2.4)	108 (1.3)		174 (2.7)	241 (1.6)	
Hispanic	303 (2.4)	115 (1.4)		151 (2.3)	267 (1.8)	
White	11 903 (92.3)	7992 (95.0)		6044 (92.8)	13 851 (93.6)	
Other^b^	156 (1.2)	103 (1.2)		64 (1.0)	195 (1.3)	
History of AF	257 (2.0)	396 (4.7)	<.001	195 (3.0)	458 (3.1)	.68
Hypertension	9286 (71.1)	6678 (78.6)	<.001	4936 (74.8)	11 028 (73.7)	.09
Hyperlipidemia	9879 (75.6)	6600 (77.7)	<.001	4842 (73.4)	11 637 (77.8)	<.001
History of MI						
None	9214 (70.5)	5893 (69.3)	.18	4856 (73.6)	10 251 (68.5)	<.001
Past	1887 (14.4)	1267 (14.9)		758 (11.5)	2396 (16.0)	
Recent	1967 (15.1)	1339 (15.8)		987 (15.0)	2319 (15.5)	
CHF	3290 (25.2)	2709 (31.9)	<.001	2358 (35.8)	3614 (24.4)	<.001
PVD	1713 (13.1)	1340 (15.8)	<.001	923 (14.0)	2130 (14.2)	.63
Diabetes	3685 (28.2)	2343 (27.6)	.31	1904 (28.9)	4124 (27.6)	.05
History of severe COPD	244 (1.9)	281 (3.3)	<.001	172 (2.6)	353 (2.4)	.28
Smoking history						
Never	6379 (51.6)	3948 (50.7)	<.001	3638 (58.8)	6689 (.48.0)	<.001
Past	4464 (36.1)	3103 (39.9)		1994 (32.2)	5573 (40.0)	
Current	1510 (12.2)	733 (9.4)		554 (9.0)	1689 (12.1)	
Creatinine level, mean (SD), mg/dL	1.1 (0.7)	1.2 (0.7)	<.001	1.0 (0.7)	1.2 (0.7)	<.001
Procedure						
CABG	6763 (51.8)	3381 (39.8)	<.001	2311 (35.0)	7833 (52.3)	<.001
AVR	3267 (25.0)	2237 (26.3)		2164 (32.8)	3340 (22.3)	
MVR	1295 (9.9)	1041 (12.3)		980 (14.9)	1356 (9.1)	
CABG and AVR	1380 (10.6)	1388 (16.3)		875 (13.3)	1893 (12.7)	
CABG and MVR	364 (2.8)	452 (5.3)		271 (4.1)	545 (3.6)	
Emergent surgery	356 (2.7)	291 (3.4)	.003	213 (3.2)	434 (2.9)	.19
Hospital 2	5041 (38.6)	3928 (46.2)	<.001	2491 (37.7)	6478 (43.3)	<.001
ACE inhibitors or ARB within 48 h preceding surgery	1728 (13.4)	1172 (14.0)	.22	805 (12.4)	2095 (14.1)	.001
β-Blockers within 24 h preceding surgery	7765 (60.0)	5222 (62.1)	.002	3790 (58.2)	9197 (62.0)	<.001

Abbreviations: ACE, angiotensin-converting enzyme; AF, atrial fibrillation; ARB, angiotensin II receptor blockers; AVR, aortic valve replacement; BMI, body mass index (calculated as weight in kilograms divided by height in meters squared); CABG, coronary artery bypass graft surgery; CHF, congestive heart failure; COPD, chronic obstructive pulmonary disease; MI, myocardial infarction; MVR, mitral valve replacement; NA, not applicable; poAF, postoperative atrial fibrillation; PVD, peripheral vascular disease.

SI conversion factor: To convert creatinine to micromoles per liter, multiply by 88.4.

^a^ *P* values are based on *t* tests for continuous variables and χ^2^ tests for categorical variables.

^b^ The other race category included American Indian, Alaska Native, Native Hawaiian, or Other Pacific Islander individuals as well as those who explicitly identified as other and those who identified as 2 or more races.

### Statistical Analysis

Descriptive statistics for covariates were calculated separately for those with and without poAF as well as separately for women and men as mean values with SDs for continuous variables or as frequencies and percentages for categorical variables. Pearson χ^2^ tests for categorical variables and Student *t* tests for continuous variables were used to examine associations between poAF status and each of the covariates as well as between sex and each of the covariates.

A logistic regression model including all measured covariates that prior literature indicated may serve as potential confounders was then used to estimate the adjusted association between sex and the odds of poAF. Predicted probabilities (assessed at all covariates held at their means, which for categorical covariates is equivalent to adjusting estimates for sample composition) were used to further elucidate the findings of logistic regression.

To assess sex-specific associations between poAF and mortality, the Cox proportional hazards model was then estimated, including sex, poAF, sex by poAF interaction, and all covariates. Covariate-adjusted Kaplan-Meier curves for the 4 groups defined by sex and poAF status were constructed based on this model, and hazard ratios (HRs) were calculated for 3 groups defined by sex and poAF combination, with men without poAF as the reference group. Using the same model, predicted probabilities of mortality were calculated at 3 time points approximately representing 3 quartiles of study follow-up: when approximately 25% of the sample was censored or deceased (30 days), when approximately 50% of the sample was censored or deceased (5 years), and when approximately 75% of the sample was censored or deceased (10 years). For these calculations and for the Kaplan-Meier curves, all covariates were held at their overall means, which for categorical covariates is equivalent to adjusting estimates for sample composition. An additional mortality analysis (using the same Cox model described previously) was performed using March 1, 2020, as the final censoring date; this analysis confirmed that COVID-19–related deaths did not substantively change the findings (eTable 1 in Supplement 1).

Our models include all covariates that prior research or theory suggest may be important controls, regardless of their statistical significance. We performed additional analyses removing these nonsignificant covariates from the models; key findings remained unchanged (eTables 2 and 3 in Supplement 1).

To account for missing data, we performed multiple imputations by chained equations on 24 196 patients, generating 20 imputed datasets. We followed the multiple imputation then deletion approach; that is, after imputation, we omitted observations that were missing poAF data from all analyses, and we omitted observations with missing follow-up data from mortality analyses. All multivariable analyses results combine estimates from 20 datasets using Rubin rules.^33^

All statistical hypothesis tests were 2-sided; the cutoff *P* < .05 was used to establish statistical significance. Data cleaning and preparation was performed using SAS version 9.4 (SAS Institute) and Stata version 16.0 (StataCorp), and all statistical analyses were performed in Stata 16.0.

## Results

### Baseline Population

A total of 21 568 patients who underwent cardiac surgical procedures between January 1, 2002, and October 1, 2016, were eligible for the analysis and had data regarding poAF (Figure 1); 6601 were women (30.6%) and 14 967 were men (69.4%); 8499 patients (39.4%) developed poAF after open heart surgery. The mean (SD) age of the full cohort was 66.5 (12.4) years; 418 patients (1.9%) were Black, 618 (2.9%) were Hispanic, 19 895 (92.2%) were White, and 259 (1.2%) were American Indian, Alaska Native, Native Hawaiian, or Other Pacific Islander individuals; those who explicitly identified as other; and those who identified as 2 or more races. Additional baseline patient and surgery-related characteristics are summarized in Table 1.

### Association Between Sex and poAF

The unadjusted analysis demonstrated women had a slightly higher incidence of poAF compared with men (2694 [40.8%] vs 5805 [38.8%]). In multivariable logistic regression analysis, however, women were observed to have a lower risk of poAF compared with men (odds ratio [OR], 0.85; 95% CI, 0.79-0.91; *P* < .001) after adjusting for patient and surgery-related risk factors (Table 2). When translated into predicted probabilities (with all covariates held at their means), men had a 40.5% chance of experiencing poAF, while women had 37.0% chance of developing poAF.

**Table 2.** Multivariable Logistic Regression Analysis for the Occurrence of Postoperative Atrial Fibrillation After Open Heart Surgery^a^

Characteristic	OR (95% CI)	*P* value
Female sex	0.85 (0.79-0.91)	<.001
Age/10	2.19 (1.74-2.77)	<.001
(Age/10) squared	0.98 (0.96-0.99)	.01
BMI/10	1.18 (0.87-1.59)	.29
(BMI/10) squared	1.00 (0.96-1.05)	.98
Race		
Asian	0.83 (0.64-1.07)	.15
Black	0.65 (0.51-0.82)	<.001
Hispanic	0.77 (0.61-0.96)	.02
White	1 [Reference]	NA
Other^b^	1.07 (0.82-1.40)	.61
History of AF	1.68 (1.42-1.99)	<.001
Hypertension	1.17 (1.08-1.26)	<.001
Hyperlipidemia	0.93 (0.86-1.01)	.08
History of MI		
Never	1 [Reference]	NA
Past	1.08 (0.99-1.18)	.07
Recent	1.10 (1.01-1.21)	.03
CHF	1.12 (1.05-1.20)	.001
PVD	1.08 (0.99-1.18)	.06
Diabetes	0.92 (0.86-0.98)	.02
History of severe COPD	1.27 (1.06-1.53)	.01
Smoking		
Never	1 [Reference]	NA
Past	1.07 (1.01-1.15)	.03
Current	1.03 (0.92-1.14)	.64
Log creatinine level	1.17 (1.03-1.32)	.02
Log creatinine level squared	1.02 (0.93-1.13)	.64
Procedure		
CABG	1 [Reference]	NA
AVR	1.63 (1.51-1.77)	<.001
MVR	2.85 (2.54-3.19)	<.001
CABG and AVR	1.59 (1.45-1.74)	<.001
CABG and MVR	2.58 (2.22-3.01)	<.001
Emergent surgery	1.49 (1.25-1.76)	<.001
Hospital 2	1.38 (1.30-1.47)	<.001
ACE inhibitors or ARB within 48 h preceding surgery	1.03 (0.94-1.12)	.51
β-Blockers within 24 h preceding surgery	1.08 (1.01-1.15)	.03

Abbreviations: ACE, angiotensin-converting enzyme; AF, atrial fibrillation; ARB, angiotensin II receptor blockers; AVR, aortic valve replacement; BMI, body mass index; CABG, coronary artery bypass graft surgery; CHF, congestive heart failure; COPD, chronic obstructive pulmonary disease; MI, myocardial infarction; MVR, mitral valve replacement; NA, not applicable; OR, odds ratio; PVD, peripheral vascular disease.

^a^ Data from multi-institutional database with surgery dates from January 1, 2002, through October 1, 2016. McFadden pseudo *R*^2^ = 0.085; area under the curve, 0.693.

^b^ The other race category included American Indian, Alaska Native, Native Hawaiian, or Other Pacific Islander individuals as well as those who explicitly identified as other and those who identified as 2 or more races.

### Association Between poAF and Mortality in Women vs Men

**Figure 2.**
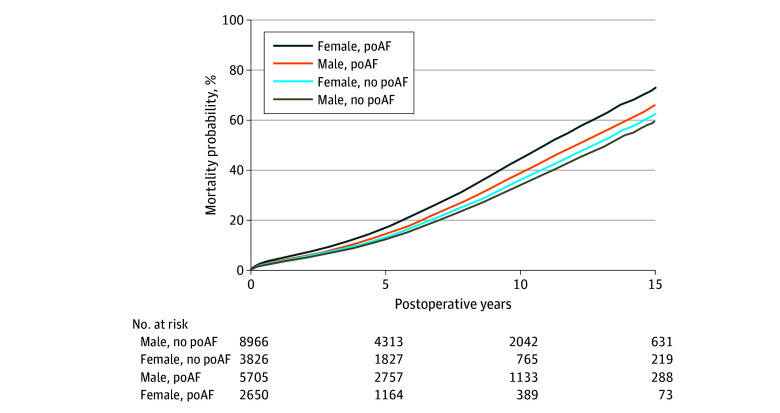
Covariate-Adjusted Mortality in All Patients Who Underwent Open Heart Surgery Between January 1, 2002, and October 1, 2016, in 2 Tertiary Care Centers, by Sex and Presence of Postoperative Atrial Fibrillation (poAF) Hazard ratios when compared with male patients without poAF are as follows: female patients without poAF: HR, 1.07 (95% CI, 0.99-1.15); male patients with poAF: HR, 1.17 (95% CI, 1.11-1.24); female patients with poAF: 1.40 (95% CI, 1.31-1.51).

The association of poAF with mortality was statistically different between women and men, as indicated by a significant interaction between poAF and sex (HR, 1.11; 95% CI, 1.02-1.23; *P* = .02) (Table 3). Men who developed poAF exhibited a higher mortality rate in comparison with men who did not experience poAF (HR, 1.17; 95% CI, 1.11-1.25, *P* < .001), while the association of poAF with mortality among women was more pronounced (HR, 1.31; 95% CI, 1.21-1.42, *P* < .001). In fact, when compared with men without poAF, women with poAF had 40% higher relative hazard of mortality (HR, 1.40; 95% CI, 1.30-1.51; *P* < .001). There was no significant difference in mortality between women and men among those without poAF (HR, 1.07; 95% CI, 1.00-1.15; *P* = .06) (Figure 2). When assessed at 10 years after surgery, with all covariates held at their means (eTable 4 in Supplement 1), men without poAF had 31.9% mortality probability, compared with men with poAF who had 36.3% mortality probability. Women without poAF had 33.7% mortality probability compared with women with poAF, who had 41.7% mortality probability. That is, with all covariates at their average values, the poAF-based gap in 10-year mortality was 4.4 percentage points among men and 8.0 percentage points among women.

**Table 3.** Cox Regression Analysis for Mortality Following Open Heart Surgery^a^

Characteristic	HR (95% CI)	*P* value
poAF	1.17 (1.11-1.24)	<.001
Female sex	1.07 (0.997-1.15)	.06
poAF and female sex interaction	1.12 (1.02-1.23)	.02
Age/10	0.66 (0.54-0.81)	<.001
(Age/10) squared	1.07 (1.06-1.09)	<.001
BMI/10	0.52 (0.43-0.63)	<.001
(BMI/10) squared	1.11 (1.08-1.14)	<.001
Race		
Asian	0.81 (0.63-1.04)	.10
Black	1.10 (0.93-1.29)	.27
Hispanic	0.86 (0.73-1.02)	.09
White	1 [Reference]	NA
Other^b^	0.94 (0.76-1.16)	.56
History of AF	1.13 (1.01-1.28)	.04
Hypertension	1.16 (1.09-1.24)	<.001
Hyperlipidemia	0.88 (0.83-0.94)	<.001
History of MI		
None	1 [Reference]	NA
Past	1.24 (1.17-1.32)	<.001
Recent	1.22 (1.15-1.31)	<.001
CHF	1.43 (1.36-1.51)	<.001
PVD	1.42 (1.34-1.50)	<.001
Diabetes	1.51 (1.44-1.59)	<.001
History of severe COPD	1.87 (1.67-2.09)	<.001
Smoking status		
Never	1 [Reference]	NA
Past	1.20 (1.14-1.27)	<.001
Current	1.43 (1.31-1.55)	<.001
Log creatinine level	1.61 (1.46-1.77)	<.001
Log creatinine level squared	1.22 (1.15-1.30)	<.001
Procedure		
CABG	1 [Reference]	NA
AVR	1.13 (1.06-1.21)	<.001
MVR	0.91 (0.82-1.02)	.10
CABG and AVR	1.28 (1.20-1.37)	<.001
CABG and MVR	1.31 (1.17-1.46)	<.001
Emergent surgery	1.31 (1.16-1.48)	<.001
Hospital 2	0.81 (0.77-0.85)	<.001
ACE inhibitors or ARB within 48 h preceding surgery	1.01 (0.95-1.07)	.78
β-Blockers within 24 h preceding surgery	1.01 (0.96-1.06)	.75

Abbreviations: ACE, angiotensin-converting enzyme; AF, atrial fibrillation; ARB, angiotensin II receptor blockers; AVR, aortic valve replacement; BMI, body mass index; CABG, coronary artery bypass graft surgery; CHF, congestive heart failure; COPD, chronic obstructive pulmonary disease; HR, hazard ratio; MI, myocardial infarction; MVR, mitral valve replacement; NA, not applicable; poAF, postoperative atrial fibrillation; PVD, peripheral vascular disease.

^a^ Data from a multi-institutional database with surgery dates from January 1, 2002, through October 1, 2016, and mortality follow up until December 1, 2022. Harrell *C* concordance index = 0.74.

^b^ The other race category included American Indian, Alaska Native, Native Hawaiian, or Other Pacific Islander individuals as well as those who explicitly identified as other and those who identified as 2 or more races.

## Discussion

In this multi-institutional retrospective cohort study, we evaluated the associations among sex, poAF, and long-term mortality after open heart surgery. We observed that men had higher incidence of poAF than women (after controls), but women who developed poAF were at higher risk of long-term mortality than men.

To our knowledge, this is the first study to examine the association among sex, poAF, and long-term mortality in a large multi-institutional population. Our findings are consistent with the results of several smaller studies that also reported men to have a greater risk of poAF,^20,33,34^ although the data in those earlier studies are not definitive, as some other studies did not detect differences according to sex,^22,23,24,25,26,27,29^ possibly due to the insufficient overall sample size or small number of female patients. Moreover, few previously published investigations evaluated the sex differences in the association of poAF with long-term patient survival, and the ones that did were smaller.^28,29^

Understanding the association among sex and the natural history of poAF as well as subsequent mortality is essential for risk stratification. Accurate prediction of poAF may allow for timely prophylaxis for those at risk of poAF and reduce the risk of poAF-associated morbidities. Clinical poAF risk prediction models to identify patients at higher risk have been developed,^35,36,37^ but these risk scores are substantially lacking due to limited generalizability, use of variables that are difficult to collect, and use of information from variables that occur after surgery or even after the onset of poAF. Elucidating the role of sex and other basic population information is imperative for developing an accurate predictive model.

The link between sex and the incidence of poAF is likely multifactorial. Even though epidemiologic sex-related characteristics of long-term AF are well described,^38^ the exact mechanisms are unknown. Recent studies revealed genetic differences between men and women in ion expression, which is important in cardiac conduction and arrhythmogenesis.^39,40,41^ However, it remains poorly understood how these differences translate into an epidemiologic risk of poAF. The association of cardiopulmonary bypass–related ischemia reperfusion, subsequent oxidative stress, and uncontrolled inflammation resulting in postoperative left atrial remodeling may also play a role in giving rise to poAF.^42,43,44^ Another plausible explanation for the lower poAF incidence in women is the beneficial effect of hormone replacement therapy in women, particularly in older age groups. Estrogen is reported to decrease the risk of common AF,^45^ possibly by reducing inflammation^46,47^ and mitigating endothelial dysfunction,^48,49^ while menstrual cycle disturbances were reported to increase the incidence of AF.^50,51^ However, the effect of hormone replacement therapy on poAF remains unclear and can be a prospective avenue for future investigations.

In addition, investigating potential associations between poAF and socioeconomic and psychosocial determinants of health is of utmost importance and can further clarify the mechanisms underlying the links among sex, poAF, and long-term mortality. While such factors are associated with common nonsurgical AF,^52,53,54^ the association between them and poAF remains unclear. Recognizing the importance of a holistic approach to each patient as well as multifactorial determinants of cardiac health, future studies should aim to investigate the role of socioeconomic factors in the pathophysiology of poAF. Taking such factors into consideration may allow clinicians to predict poAF with a higher degree of certainty and to facilitate a tailored personalized approach to each cardiac surgical patient, including preoperative risk stratification, intraoperative prophylactic management, and postoperative follow-up care, while accounting for the diversity of backgrounds. The observed elevated risk of long-term mortality in women with poAF underscores the need for refined approach to postoperative management, continuous cardiac monitoring, regular follow-ups, and proactive interventions in this patient population.

### Limitations

Our study has some limitations. First, while it is multi-institutional, the patient sample of our retrospective study was limited to the same geographical region. However, data analysis including more than 21 000 patients demonstrated consistent results from 2 different institutions that rely on separate cardiac surgical service lines. Therefore, the external validity of the results is likely credible. Second, our results are based on patients who underwent CABG, AVR, MVR as well as combined procedures and may not be applicable to the other types of operative treatment. Third, the number of patients who are younger than 50 years or older than 90 years as well as American Indian or Alaska Native, Asian, Black, Hispanic, multiracial, and Native Hawaiian or Other Pacific Islander patients and patients who identify as another race or ethnicity is limited in our sample. The validity of our results for patients in these demographic categories may be reduced. Fourth, the results of our retrospective study may be subject to residual confounding, and unmeasured variables related to sex could account for the observed differences, especially in patients undergoing CABG.^55^ In our study, we have included a wide range of open heart surgical procedures to mitigate this potential bias; still, we have to be cautious about causality attributions. Lastly, detecting poAF is challenging given that the clinical use of telemetry is often stopped prior to hospital discharge while the peak incidence of poAF falls on postoperative days 2 to 5. Regardless, this timing-related challenge is likely not sex-specific and cannot account for our findings.

## Conclusions

In this multi-institutional retrospective cohort study of cardiac surgical patients, we found that women had a lower risk of developing poAF than men (after controls); however, women who developed poAF were at higher risk of long-term mortality compared with men with poAF. Our findings suggest that women may have protective factors against the development of poAF. However, once poAF takes place, women may be more vulnerable to the associated long-term morbidities.

Future studies may elucidate the exact mechanisms responsible for the observed sex-based disparities in poAF incidence and long-term associated morbidities. Genetic, cardiac structural, hormonal, socioeconomic, and psychosocial risk factors could be the potential targets for prospective investigations. A multidomain approach to elucidating pathophysiology of poAF may enable a holistic approach to patient care while accounting for the diversity of backgrounds of women and men undergoing cardiac surgery.

## Notes

Supplement 1. **eTable 1.** Cox Regression Analysis for Mortality Following Open Heart Surgery Excluding COVID-19 Period **eTable 2.** Multivariable Logistic Regression Analysis for the Occurrence of Postoperative Atrial Fibrillation After Open Heart Surgery (Including Only Significant Covariates) **eTable 3.** Cox Regression Analysis for Mortality Following Open Heart Surgery (Including Only Significant Covariates) **eTable 4.** Predicted Mortality Probabilities at Different Time Points Based on Sex and Postoperative Atrial Fibrillation, Based on Cox Regression Analysis for Mortality Following Open Heart Surgery

Supplement 2.
Data Sharing Statement
