# Supplementary material for: Sex, Atrial Fibrillation, and Long-Term Mortality After Cardiac Surgery
Source: JAMA Netw Open. 2024 Aug 21;7(8):e2426865. doi: 10.1001/jamanetworkopen.2024.26865 (PMC11339658; doi:10.1001/jamanetworkopen.2024.26865)
Supplement: Supplement 1. — eTable 1. Cox Regression Analysis for Mortality Following Open Heart Surgery Excluding COVID-19 Period eTable 2. Multivariable Logistic Regression Analysis for the Occurrence of Postoperative Atrial Fibrillation After Open Heart Surgery (Including Only Significant Covariates) eTable 3. Cox Regression Analysis for Mortality Following Open Heart Surgery (Including Only Significant Covariates) eTable 4. Predicted Mortality Probabilities at Different Time Points Based on Sex and Postoperative Atrial Fibrillation, Based on Cox Regression Analysis for Mortality Following Open Heart Surgery [file jamanetwopen-e2426865-s001.pdf]

Supplemental Online Content

Karamnov S, Sarkisian N, Wollborn J, et al. Sex, atrial fibrillation, and long-term mortality after cardiac surgery. *JAMA Netw Open*. 2024;7(8):e2426865. doi:10.1001/jamanetworkopen.2024.26865

eTable 1. Cox Regression Analysis for Mortality Following Open Heart Surgery Excluding COVID-19 Period
eTable 2. Multivariable Logistic Regression Analysis for the Occurrence of Postoperative Atrial Fibrillation After Open Heart Surgery (Including Only Significant Covariates)
eTable 3. Cox Regression Analysis for Mortality Following Open Heart Surgery (Including Only Significant Covariates)
eTable 4. Predicted Mortality Probabilities at Different Time Points Based on Sex and Postoperative Atrial Fibrillation, Based on Cox Regression Analysis for Mortality Following Open Heart Surgery

This supplemental material has been provided by the authors to give readers additional information about their work.

© 2024 Karamnov S et al. JAMA Network Open.

eTable 1. Cox Regression Analysis Predicting Mortality Following Open Heart Surgery Excluding COVID-19 Period[a]

Characteristic	HR	(95% CI)	P value
poAF	1.18	(1.11-1.25)	<0.001
Female sex	1.09	(1.02-1.18)	0.02
poAF and female sex interaction	1.12	(1.01-1.23)	0.04
Age/10	0.62	(0.50-0.76)	<0.001
(Age/10) squared	1.08	(1.06-1.09)	<0.001
BMI/10	0.49	(0.41-0.60)	<0.001
(BMI/10) squared	1.11	(1.08-1.15)	<0.001
Race			
Asian	0.83	(0.64-1.09)	0.18
Black	1.03	(0.86-1.23)	0.73
Hispanic	0.86	(0.72-1.03)	0.1
White	1 [Reference]		NA
Other [b]	0.88	(0.70-1.11)	0.27
History of AF	1.13	(1.00-1.28)	0.06
Hypertension	1.17	(1.09-1.25)	<0.001
Hyperlipidemia	0.87	(0.81-0.93)	<0.001
History of MI			
None	1 [Reference]		NA
Past	1.23	(1.15-1.31)	<0.001
Recent	1.22	(1.14-1.31)	<0.001
CHF	1.44	(1.36-1.52)	<0.001
PVD	1.42	(1.34-1.51)	<0.001
Diabetes	1.52	(1.44-1.60)	<0.001
History of severe COPD	1.95	(1.74-2.19)	<0.001
Smoking status			
Never	1 [Reference]		NA
Past	1.20	(1.14-1.27)	<0.001
Current	1.40	(1.28-1.53)	<0.001
Log creatinine level	1.63	(1.47-1.80)	<0.001
Log creatinine level squared	1.22	(1.15-1.30)	<0.001
Procedure			
CABG	1 [Reference]		NA
AVR	1.10	(1.03-1.19)	0.007
MVR	0.89	(0.80-1.00)	0.04
CABG and AVR	1.27	(1.18-1.36)	<0.001
CABG and MVR	1.31	(1.17-1.47)	<0.001
Emergent surgery	1.31	(1.15-1.49)	<0.001
Hospital 2	0.81	(0.77-0.85)	<0.001

© 2024 Karamnov S et al. JAMA Network Open.

ACE inhibitors or ARB within 48 h preceding surgery	1.02	(0.95-1.09)	0.58
B-blockers within 24 h preceding surgery	1.00	(0.94-1.05)	0.86

Abbreviations: ACE, angiotensin-converting enzyme; AF, atrial fibrillation; ARB, angiotensin II receptor blockers; AVR, aortic valve replacement; BMI, body mass index; CABG, coronary artery bypass graft surgery; CHF, congestive heart failure; COPD, chronic obstructive pulmonary disease; HR, hazard ratio; MI, myocardial infarction; MVR, mitral valve replacement; NA, not applicable; poAF, postoperative atrial fibrillation; PVD, peripheral vascular disease.

[a] Data from a multi-institutional database with surgery dates from January 1, 2002, through October 1, 2016, and mortality follow up until March 1, 2020. Harrell C concordance index = 0.74.

[b] The other race category included American Indian, Alaska Native, Native Hawaiian, or Other Pacific Islander individuals as well as those who explicitly identified as other and those who identified as 2 or more races.

© 2024 Karamnov S et al. JAMA Network Open.

eTable 2. Multivariable Logistic Regression Analysis for the Occurrence of Postoperative Atrial Fibrillation After Open Heart Surgery (Including Only Significant Covariates)[a]

Characteristic	OR	(95% CI)	P value
Female sex	0.86	(0.80-0.92)	<0.001
Age/10	2.24	(1.78-2.83)	<0.001
(Age/10) squared	0.97	(0.96-0.99)	0.004
Race			
Asian	0.78	(0.60-1.01)	0.06
Black	0.65	(0.51-0.82)	<0.001
Hispanic	0.76	(0.60-0.95)	0.02
White	1 [Reference]		NA
Other [b]	1.05	(0.80-1.36)	0.74
History of AF	1.71	(1.44-2.02)	<0.001
Hypertension	1.19	(1.10-1.28)	<0.001
History of MI			
None	1 [Reference]		NA
Past	1.08	(0.99-1.18)	0.08
Recent	1.10	(1.01-1.21)	0.03
CHF	1.13	(1.05-1.21)	0.001
Diabetes	0.95	(0.89-1.02)	0.16
History of severe COPD	1.29	(1.07-1.55)	0.007
Smoking			
Never	1 [Reference]		NA
Past	1.08	(1.01-1.15)	0.02
Current	1.02	(0.92-1.13)	0.70
Log creatinine level	1.20	(1.10-1.31)	<0.001
Procedure			
CABG	1 [Reference]		NA
AVR	1.66	(1.53-1.80)	<0.001
MVR	2.77	(2.48-3.10)	<0.001
CABG and AVR	1.60	(1.46-1.75)	<0.001
CABG and MVR	2.54	(2.18-2.95)	<0.001
Emergent surgery	1.51	(1.27-1.79)	<0.001
Hospital 2	1.38	(1.30-1.47)	<0.001
β-blockers within 24 h preceding surgery	1.08	(1.01-1.15)	0.02

Abbreviations: AF, atrial fibrillation; AVR, aortic valve replacement; CABG, coronary artery bypass graft surgery; CHF, congestive heart failure; COPD, chronic obstructive pulmonary disease; MI, myocardial infarction; MVR, mitral valve replacement; NA, not applicable; OR, odds ratio.

[a] Data from multi-institutional database with surgery dates from January 1, 2002, through October 1, 2016. McFadden pseudo R^2 = 0.082; area under the curve, 0.692.

[b] The other race category included American Indian, Alaska Native, Native Hawaiian, or Other Pacific Islander individuals as well as those who explicitly identified as other and those who identified as 2 or more races.

© 2024 Karamnov S et al. JAMA Network Open.

eTable 3. Cox Regression Analysis for Mortality Following Open Heart Surgery (Including Only Significant Covariates)[a]

Characteristic	HR	(95% CI)	P value
poAF	1.17	(1.11-1.24)	<0.001
Female sex	1.07	(1.00-1.15)	0.06
poAF and female sex interaction	1.12	(1.01-1.23)	0.03
Age/10	0.66	(0.54-0.80)	<0.001
(Age/10) squared	1.07	(1.06-1.09)	<0.001
BMI/10	0.53	(0.44-0.63)	<0.001
(BMI/10) squared	1.11	(1.08-1.14)	<0.001
History of AF	1.14	(1.01-1.28)	0.03
Hypertension	1.16	(1.09-1.24)	<0.001
Hyperlipidemia	0.88	(0.83-0.94)	<0.001
History of MI			
None	1 [Reference]		NA
Past	1.24	(1.17-1.32)	<0.001
Recent	1.22	(1.14-1.30)	<0.001
CHF	1.43	(1.36-1.51)	<0.001
PVD	1.42	(1.34-1.50)	<0.001
Diabetes	1.51	(1.43-1.59)	<0.001
History of severe COPD	1.87	(1.67-2.09)	<0.001
Smoking status			
Never	1 [Reference]		NA
Past	1.20	(1.14-1.27)	<0.001
Current	1.44	(1.33-1.56)	<0.001
Log creatinine level	1.60	(1.46-1.77)	<0.001
Log creatinine level squared	1.22	(1.15-1.30)	<0.001
Procedure			
CABG	1 [Reference]		NA
AVR	1.13	(1.06-1.21)	<0.001
MVR	0.92	(0.82-1.02)	0.11
CABG and AVR	1.28	(1.20-1.37)	<0.001
CABG and MVR	1.30	(1.17-1.45)	<0.001
Emergent surgery	1.31	(1.16-1.49)	<0.001
Hospital 2	0.81	(0.77-0.85)	<0.001

Abbreviations: AF, atrial fibrillation; AVR, aortic valve replacement; BMI, body mass index; CABG, coronary artery bypass graft surgery; CHF, congestive heart failure; COPD, chronic obstructive pulmonary disease; HR, hazard ratio; MI, myocardial infarction; MVR, mitral valve replacement; NA, not applicable; poAF, postoperative atrial fibrillation; PVD, peripheral vascular disease.
[a] Data from a multi-institutional database with surgery dates from January 1, 2002, through October 1, 2016, and mortality follow up until December 1, 2022. Harrell C concordance index = 0.74.

© 2024 Karamnov S et al. JAMA Network Open.

eTable 4. Predicted Mortality Probabilities (%) at Different Timepoints Based on Sex and Postoperative Atrial Fibrillation, Based on Cox Regression Analysis for Mortality Following Open Heart Surgery[a]

Censoring Timepoint	Patient Sample Censored/Deceased (%)	Predicted Probability of Mortality (%)			
		Men no poAF	Men poAF	Women no poAF	Women poAF
30 days	25.2	1.0	1.2	1.1	1.4
5 years	51.9	11.6	13.5	12.4	15.9
10 years	78.8	31.9	36.3	33.7	41.7

Abbreviation: poAF, postoperative atrial fibrillation.
[a] Data from multi-institutional database with surgery dates from January 1, 2002, through October 1, 2016, and mortality follow up until December 1, 2022. All the covariates held at their sample means.

© 2024 Karamnov S et al. JAMA Network Open.
