# Supplementary material for: Sex, Atrial Fibrillation, and Long-Term Mortality After Cardiac Surgery
Source: JAMA Netw Open. 2024 Aug 21;7(8):e2426865. doi: 10.1001/jamanetworkopen.2024.26865 (PMC11339658; doi:10.1001/jamanetworkopen.2024.26865)
Supplement: Supplement 2. — Data Sharing Statement [file jamanetwopen-e2426865-s002.pdf]

Data Sharing Statement

Karamnov. Sex, Atrial Fibrillation, and Long-Term Mortality After Cardiac Surgery. *JAMA Netw Open*. Published August 21, 2024. doi:10.1001/jamanetworkopen.2024.26865

Data
Data available: No

Additional Information
Explanation for why data not available: Data was obtained retrospectively after IRB approval with a waiver of informed consent. As patients did not consent for the data to be shared, we cannot share the data.
